# How Do Brazilian Consumers Understand Food Groups in the Food-based Dietary Guidelines?

**DOI:** 10.3390/foods13020338

**Published:** 2024-01-21

**Authors:** Jordanna Santos Monteiro, Eduardo Yoshio Nakano, Renata Puppin Zandonadi, Raquel Braz Assunção Botelho, Wilma Maria Coelho Araújo

**Affiliations:** 1 Department of Nutrition, School of Health Sciences, University of Brasilia (UnB), Campus Darcy Ribeiro, Asa Norte, Brasilia 70910-900, Brazil; jordanna.santosmonteiro@gmail.com (J.S.M.); renatapz@unb.br (R.P.Z.); raquelbotelho@unb.br (R.B.A.B.); 2 Department of Statistics, Central Institute of Sciences, University of Brasilia (UnB), Campus Darcy Ribeiro, Asa Norte, Brasilia 70910-900, Brazil; nakano@unb.br

**Keywords:** food classification, food group, Food-based Dietary Guidelines, animal kingdom, plant kingdom, fruits, vegetables, cereals, and legumes, fats, sugar

## Abstract

In the Food-based Dietary Guidelines (FBDGs), food classification is based on food groups and nutrient sources. Much research has already investigated multiple aspects of consumer understanding of the information described in these documents. However, no study has evaluated consumer understanding of all food items contained in the groups described in the FBDGs. This study aimed to assess Brazilian consumers’ understanding of food classification according to food groups in the concepts of the FBDGs. Therefore, an instrument, Consumer Understanding of Food Groups (UFG), was constructed and validated to assess consumer understanding of food groups. The instrument comprised 44 items approved by experts (agreement > 80%). A total of 894 Brazilians from all regions participated in this study. The results suggest that 48.9% of the participants believe it is easier to classify food according to food groups. The classification of food groups is based on the origin of the food (animal and vegetable). Although consumers easily recognize foods according to their origin, we still identify asymmetries regarding including food items from the animal kingdom and species from the plant kingdom. This exploratory study highlights important information that can contribute to improving the FBDGs. It is essential to consider consumers’ understanding and guide them regarding choices from a technical point of view.

## 1. Introduction

Dietary guidelines guide consumer behavior based on national food, nutrition, and health policies. They consider the aspect of healthy eating and must incorporate scientific principles [1]. It is known that diet is a major lifestyle determinant of health [2,3,4]. Improving dietary habits by reducing sodium intake and increasing whole grain and fruit intake could significantly decrease morbidity and mortality from noncommunicable diseases. Further, limiting sugar and saturated fatty acids and increasing fiber and unsaturated fatty acid intake also benefit public health [2,5]. As a diet in line with the current evidence-based guidelines decreases the risk of all-cause and cause-specific mortality, it is crucial to guide food choices based, simultaneously, on scientific knowledge and consumer understanding of such choices [2,3,6,7,8].

In this scenario, classifying foods based on their physical, chemical, nutritional, and biological characteristics and other food components is a strategy for developing programs and policies in the nutrition, health, agriculture, and food industry fields [9,10,11,12,13]. Food classification is a distribution that lists different foods in groups, which may or may not contain subgroups, defined based on common properties and mainly identified by the consumer. This grouping identifies a collection of food items that are not generally considered variants of the same food but share characteristics regarding nature, origin, or use [14,15,16].

According to the Food and Agriculture Organization (FAO) [17], the food classification used in the Food-based Dietary Guidelines (FBDGs) is based on food groups and nutrient sources. The food classification according to food groups (cereals, fruits, vegetables, dairy products, meat, among others) categorizes different foods according to their origin, nutritional properties, and marketing characteristics [6,16]. Thus, the FBDGs provide evidence-based, practical, and actionable recommendations that aim to guide the dietary behaviors of a nation through consumer education and targeted health policies and programs. The placement and classification of specific foods within the dietary guidelines are based on the traditional dietary pattern of a country [18,19].

Considered food and nutritional education instruments for a population, FBDGs must describe foods with appropriate, understandable nomenclature specific to each country, and they must consider the classification of foods from a scientific point of view and “translate” this information to the consumer, avoiding inconsistencies and ambiguous information [18,19,20,21,22]. Researchers have already investigated consumer understanding of graphic information and information described in the FBDGs, as well as the consumption of specific food groups contained in these guidelines, such as the dairy and vegetable groups, and comparison of information between the FBDGs of some countries [1,6,18,23,24,25,26,27,28,29,30,31,32]. However, no study has assessed consumer understanding of all food items contained in their groups and described in the FBDGs available on the FAO website [17]. Therefore, this study aimed to assess Brazilian consumers’ understanding of food classification according to food groups and the Food-based Dietary Guideline concepts.

## 2. Materials and Methods

### 2.1. Study Design

This qualitative–quantitative, cross-sectional study was performed with Brazilian consumers. The first step was developing and validating the Consumer Understanding of Food Groups (UFG) instrument. The instrument validation was conducted according to Boateng et al. [33]. The UFG underwent content and semantic analysis. The University of Brasília Ethics Committee approved this project (38084620.1.0000.8093). After validation, the UFG was sent electronically to consumers living in all geographic regions of Brazil.

### 2.2. Construction of the Understanding of Food Groups (UFG) Instrument

The items proposed for the instrument were prepared according to the nomenclature and food classification described in the 89 Food-based Dietary Guidelines available on the FAO website [17]. The preliminary version comprised 66 items (constructs).

**Figure 1:**
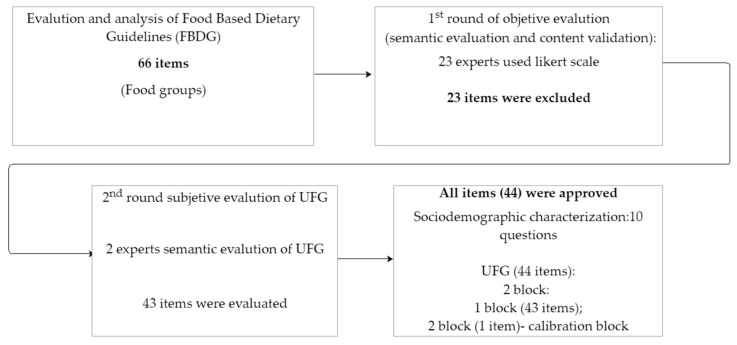
Stages of the construction, content validation, and semantic evaluation of “Understanding of Food Groups” (UFG).

Fifty-nine experts (university professors, food technologists, food scientists, and nutritionists) were invited to evaluate the items’ clarity and relevance. They were also asked to include their suggestions to modify or include an item, if necessary. Twenty-three experts agreed to participate in this part of the study and used the following criteria to evaluate the items: (i) Item clarity was assessed using a 5-point scale: 1 (I did not understand at all), 2 (I understood a little), 3 (I understood almost everything, with reservations), 4 (I understood almost everything), and 5 (I completely understood). (ii) Item relevance was assessed using a 5-point scale: 1 (inadequate), 2 (very little adequate), 3 (little adequate), 4 (adequate), and 5 (very adequate). Data were analyzed considering each evaluated item and the suggestions presented for the item’s reformulation. Two university researchers in food science and technology and nutrition (W.M.C.A.; J.S.M) evaluated the items and modified them according to the experts’ suggestions. The criterion for keeping the item in the instrument was to obtain at least 80% agreement between the experts on each item. Items that did not reach 80% were reworked and re-evaluated. If suggested by experts, the item was excluded (Figure 1).

The final UFG version comprised 43 food items and food groups. The food items were described according to the information available in the FBDGs, and some only had images of the food or items that represented a specific group. This is because the FBDGs provide evidence-based, practical, and actionable recommendations to guide dietary behavior [34]. The respondents were asked to answer about food and food groups by classifying the items as “True”, “False”, or “I do not know” (Table A1; Appendix A). To analyze the results, we considered the percentages of “True”, “False”, and “I do not know” responses. We compared these percentages to sociodemographic data and the respective item’s botanical, agronomic, and nutritional characteristics. Also, we added one item to identify why the consumer considered it “to be easier” to classify food (1) “level of processing”, (2) food groups, (3) nutrient sources, and (4) list of ingredients, if this was the consumer’s answer to food classification, previously validated and applied to the Brazilian population [6] (Table A2; Appendix A). Sociodemographic data were added, such as nationality, administrative region, state residence, age, gender, education level, and monthly income.

The UFG was distributed nationwide using a convenience sample with the snowball method [33]. The validation process of an instrument requires 20 respondents per item (20:1) [35]. Therefore, to validate the UFG, the minimum sample size was estimated at 860 (20:43) participants. The instrument was applied using the Google Forms™ (Menlo Park, CA, USA) platform to a convenience sample of adults from all Brazilian regions. Participants were recruited using social media advertising (Facebook™ Menlo Park, CA, USA, Instagram™ Menlo Park, CA, USA, and WhatsApp™ Menlo Park, CA, USA). The data collection occurred from February to December 2022.

### 2.3. Statistical Analysis

Categorical variables (sociodemographic characteristics) were described as frequencies (*n*) and percentages (%), and quantitative variables were described as mean and standard deviation or standard error. Independent Student’s t-test, ANOVA with Tukey’s post hoc tests, and ANOVA with Tukey’s post hoc tests were used to examine differences in scores. The chi-square test was used to compare categorical variables. The level of statistical significance was set at 5% (*p* < 0.05). The statistical software IBM SPSS Statistics for Windows version 22 (IBM Corp, Armonk, NY, USA) was used for the analysis.

## 3. Results

### Understanding of Food Groups (UFG): Application

Of the 902 individuals who accessed the UFG, 99.1% (*n* = 894) agreed to participate in this study and signed the Free and Informed Consent Form. The participants were mostly from the Southeast Brazilian region (*n* = 348; 38.92%), followed by the Northeast (*n* = 208; 23.2%), North (*n* = 120; 13.4%), Midwest (*n* = 118; 13.2%) and South (*n* = 100; 11.2%) (Appendix B). Most respondents were female (*n* = 559; 62.5%), and more than half were over 40 y/o (*n* = 528; 59%; *n* = 528). Most respondents had an educational level equivalent to postgraduation (*n* = 683; 76.4%), and most respondents had an individual income above 10 minimum wages (*n* = 353; 39.5%) (Appendix B).

In Table A1 (Appendix A), the UFG items are described: 12 items refer to food from the meat and meat products group, 8 items refer to the dairy products group, 12 items refer to the cereals, legumes, and vegetables group, and 11 items refer to the vegetables and fruits group, according to the FBDGs [17]. Data for the frequency of responses indicated as “True”, “False” and “I don’t know”, relating to Block 1, are described in Appendix C. Of the 43 items, only 9 (21%) were indicated as “True” with a frequency greater than 90%. Of these, 98.7% (*n* = 882), 98.3% (*n* = 879), 99.2% (*n* = 887), and 94.3% (*n* = 843) of respondents indicated items I-15, I-19, I-32, and I-40 (curd, yogurt, cheeses, and cottage cheese), respectively, as belonging to the dairy products group. Items I-18 (sweet potatoes, carrots, cassava, and turnips are roots) (*n* = 832; 93.1%); I-20 (coconut and avocado are fruits) (*n* = 823; 92.1%); I-31 (rabbit, wild boar, snake, and alligator meats are game meats) (*n* = 808; 90.4%), I-37 (lettuce, arugula, and kale are leafy foods) (*n* = 865; 96.8%), and I-42 (oils, olive oils, butter, and margarine are fats and oils) (*n* = 842; 94.2%) also had a frequency above 90% of responses indicated as “True”. More than 90% of respondents indicated two items as “False”: the I-22 (nuts, chestnuts, and seeds are “vegetables”) (*n* = 825; 92.3%) and I-24 (seafood, poultry, vegetables, eggs, and dairy products are meat) (*n* = 807; 90.3%) (Table 1).

The sociodemographic data associated with the items referring to the meat group, the frequency of responses indicated as “True” (Appendix C), and the degree of significance are described in Table 2. Of the 12 items in the meat group, 9 showed significant differences (*p* < 0.05) for the variables gender, age group, geographic region, education level, and income. For gender, I-1 and I-31 showed significant differences between the answers indicated as “True”: female respondents indicated that “meat is any edible part of animals (*n* = 257, 46%) and rabbit, wild boar, snake, alligator meat are game meats (*n* = 520, 93%)”. A significant difference was also observed for the age group concerning I-43 (meats are classified into beef, pork, sheep, goat, buffalo, poultry, fish, amphibians, mammals, and reptiles): more than 70% (*n* = 373) of respondents over 40 years old indicated the item as “True” (Table 2).

Items I-9 and I-34, associated with the fish and fish group, also indicated significant differences in age group, geographic region, education level, and income. Item I-9 presented the highest percentage of responses marked as “False” (*n* = 720, 80.5%). Respondents from the Northeast region (*n* = 20, 9.6%), with higher education (*n* = 21, 12.5%), and with income of up to four minimum wages (*n* = 24, 10.8%) were those who indicated “seaweed is a seafood (I-9)” as “True”. For item I-34 (fresh and saltwater fish), respondents over 40 years (*n* = 470, 89%) who did not report income (*n* = 39, 90.7%) provided a higher percentage of responses indicated as “True” (Table 2).

Regarding items I-11 (meat is beef) and I-24 (seafood, poultry, vegetables, eggs, and dairy products are meat), a significant difference in the level of education and income variables was identified. For item I-11, 770 (86.1%) respondents indicated the item as “False”. Respondents with higher education (*n* = 32, 19%) and an income of up to four minimum wages (*n* = 41, 18.5%) were those who indicated the highest percentage of responses as “True” for this item. Regarding item I-24, a higher percentage of respondents indicated the item as “False” (*n* = 807, 90.3%). Respondents with a level of education up to high school (*n* = 5, 11.6%) and who did not report an income (*n* = 4, 9.3%) were those who indicated a higher percentage of responses marked as “True” for this item (Table 2).

Items I-5 and I-21, associated with the meat products group, showed significant differences in gender, age group, and geographic region. Male respondents (*n* = 306, 91.3%) indicated item I-5 (hot dogs, sausage, ham, and mortadella, among others, are meat derivatives) as “True”, as did respondents over 40 years (*n* = 478, 90.5%) and residents in the South region of Brazil (*n* = 93, 93%). Among male respondents, 170 (50.7%) indicated item I-21 (hot dogs, sausage, ham, and mortadella, among others, are meats) as “True”, as did residents in the South region (*n* = 51; 51%) (Table 2).

The frequency of responses indicated as “True” (Block 1), the degree of significance, and the sociodemographic data associated with the items referring to the dairy products group are described in Table 2. Of the eight items described in this group, there was a significant difference (*p* < 0.05) for five of them concerning the variables: gender, age group, geographic region, education level, and income. There was a significant difference in I-8 (kefir is a dairy product) and I-23 (tofu is a cheese) regarding the frequency of responses indicated as “True” for the variables gender, age group, and geographic region. Female respondents (*n* = 237, 42.4%; *n* = 248, 44.4%, respectively) and those over 40 years (*n* = 201, 38.1%; *n* = 239, 45.3%, respectively) indicated items I-8 and I-23 as “True”. Respondents living in the South region (*n* = 47, 47%) indicated a higher percentage of responses as “True” for items I-8 to I-23. The highest percentage of items indicated as “True” was in the North region (*n* = 60, 50%) (Table 2).

Significant differences existed for I-19, I-13, and I-40 and all variables. Female respondents (*n* = 553, 98.9%), over 40 years (*n* = 525, 99.4%), with a level of education up to high school (*n* = 43, 100%), and who did not inform their income (*n* = 43, 100%), indicated a higher percentage of items as “True” for I-19 (curd is a derivative of milk). Female respondents (*n* = 216, 38.6%), up to 39 years (*n* = 144, 39.3%), living in the North region (*n* = 54, 45%), with higher education (*n* = 73, 43.5%), and with an income of up to four minimum wages (*n* = 98, 44.1%), indicated a higher percentage of items as “True” for I-13 (soy milk and rice milk are milk). For item I-40 (yogurt, cheese, and cottage cheese are dairy products), there was a higher frequency of items indicated as “True” for respondents over 40 years (*n* = 507, 96%) and with education at the postgraduate level (*n* = 652, 95.5%) (Table 2).

The sociodemographic data associated with the items referring to the cereals, legumes, and vegetables group, with the frequency of responses indicated as “True” (Block 1), and the degree of significance are described in Table 3. For 10 of the 12 items described in the cereals, legumes, and vegetables group, there was a significant difference (*p* < 0.05) for the variables gender, age group, geographic region, education level, and income. Male respondents indicated a higher frequency of items as “True” for I-12 (oats, rice, wheat, corn, and cereal seeds; *n* = 303; 90.4%) and I-35 (gnocchi, pasta, lasagna, and cannelloni are derived from cereals; *n* = 209, 62.4). A higher frequency of items indicated as “True” was observed for I-39 (corn, as grain or on the cob, wheat grains, and other cereals are part of the group of cereals and legumes) in females (*n* = 329, 58.9%) (Table 3).

There were significant differences in the education level and income variables for I-2 (green banana is a type of cereal). Respondents with an education level of up to high school (*n* = 4, 9.3%) and with an income of up to four minimum wages (*n* = 15, 6.8%) indicated a greater frequency of answers as “True”. For I-33 (soy and chickpeas are “legumes”), there was a significant difference in the gender and age group variables. Male respondents (*n* = 104, 31%) and those over 40 years (*n* = 162, 30.7%) indicated a greater frequency of answers as “True”. For I-14 (soy steak is a type of meat), significant differences were observed for the geographic region, education level, and income variables. Respondents living in the Central-west region (*n* = 30, 25.4%), with higher education (*n* = 41, 24.4%), and with an income of up to four minimum wages (*n* = 47, 21.2%) indicated a greater frequency of answers as “True” (Table 3).

Respondents indicated a higher frequency of responses as “True” (*n* = 666; 74.5%) for I-41 (beans of all colors, fava beans, lentils, and peanuts are legume seeds) than for items that describe such foods as cereals or vegetables or legumes. There were significant differences for all variables for I-16, I-27, I-36, and I-41 relating to the legume group (Table 3).

Male respondents (*n* = 175, 52.2%; *n* = 222, 66.3%, respectively) and those over 40 years old (*n* = 89, 54.7%; *n* = 340, 64.4%, respectively) indicated a higher frequency of answers as “True” for I-16 (beans, soy, lentils, and chickpeas are cereals) and I-27 (beans, soy, lentils, and chickpeas are vegetables). For I-27, there was also a significant difference in the education level and income variables. Respondents with postgraduate degrees (*n* = 421, 61.6%), as well as those who did not inform their income (*n* = 28, 65.1%), indicated a greater frequency of answers as “True” (Table 3).

There were significant differences for I-36 (beans, soy, lentils, chickpeas, and legumes) for the geographic region, education level, and income variables. There were significant differences in the age group and geographic region variables for I-41 (beans of all colors, fava beans, lentils, and peanuts are legume seeds). Residents of the South region indicated a higher frequency of responses as “True” for items I-36 and I-41 at 73% and 85%, respectively. Respondents with a postgraduate degree (*n* = 485, 71%) and with an income greater than 10 minimum wages (*n* = 261, 73.9%) indicated a greater frequency of answers as “True” for I-36. For I-41, respondents aged over 40 years (*n* = 410, 77.7%) indicated a greater frequency of answers as “True” (Table 3).

The sociodemographic data associated with the items referring to the fruits, oils, fats, and vegetables group, the frequency of responses indicated as “True” (Appendix C), and the degree of significance are described in Table 3. Regarding the 11 items from the fruit, oils, fats, and vegetables group, for eight items, there was a significant difference (*p* < 0.05) in all variables. Of the two items related to fruit (I-7 and I-20), only one significantly differed for the geographic region variable. Respondents from the Southeast region (*n* = 305, 87.6%) indicated a higher frequency of responses as “True” for I-7 (tomato, melon, watermelon, pepper, and bell peppers are fruits). Female respondents (*n* = 453, 81%) indicated a greater frequency of responses as “True” for I-6 (garlic and onion are bulbs). In contrast, male respondents (*n* = 199, 59.4%) indicated a greater frequency of answers as “True” for I-10 (vegetables are leaves, flowers, fruits, stems, seeds, tubers, and roots). For I-10, there was also a significant difference in the geographic region variable; residents of the Central-west region indicated a greater frequency of responses as “True” (*n* = 82, 69.5%). For items I-4 (cauliflower, broccoli, and artichoke are flowers), I-6 (garlic and onions are bulbs), I-22 (nuts, chestnuts, and seeds are vegetables), I-25 (“vegetables” are the edible green leafy parts of plants), and I-37 (lettuce, arugula, and kale are leafy foods), there was a significant difference in the age group variable. For all these items, respondents aged over 40 years indicated a greater frequency of responses as “True” (67%, 85.2%, 5.7%, 66.7%, and 97.2%, respectively) (Table 3).

For items I-6 and I-25, it was found that there was also a significant difference in the geographic region and income variables. Residents of the South region indicated a higher frequency of responses as “True” at 84% and 64%, respectively. For I-6, it was observed that respondents who did not inform their income (*n* = 37, 86%) indicated a greater frequency of answers as “True”. For I-25, respondents with an income greater than 10 minimum wages (*n* = 226, 64%) indicated a greater frequency of responses as “True” (Table 3).

There were significant differences in the education level variable for items I-4, I-18 (sweet potatoes, carrots, cassava, and turnips are roots), I-22, and I-25. Respondents with a postgraduate level education indicated a greater frequency of answers as “True” for I-18 (*n* = 641, 93.9%), I-22 (*n* = 31, 4.5%), and I-25 (*n* = 421, 61.6%). Respondents who studied until high school indicated a greater frequency of answers as “True” for I-4 (*n* = 34, 79.1%) (Table 3).

Regarding the data from Block 2, to identify why the consumer considered it “easier” to classify foods, whether by (1) the “processing level”, (2) food groups, (3) nutrient sources, or (4) the list of ingredients, there was a significant difference only between respondents in the age group variable. In general, the results suggested that most respondents believed that it was “easier” to classify foods according to food groups (*n* = 437; 48.9%), with a significant difference in respondents aged over 40 years (*n* = 270; 51.1%).

## 4. Discussion

This study, with respondents from all Brazilian states, is the first to evaluate consumer understanding regarding the classification of foods according to food groups, described in the Food-based Dietary Guidelines on the FAO website [17]. Our UFG validation followed Boateng et al. [33]. Regarding the number of experts needed to assess the suitability of each item, there is no consensus in the literature on this number [33,36,37]. The number of respondents was equal to 894, which follows Hair et al.’s [34] recommendation for validating an instrument with a minimum number of twenty respondents per item.

In this study, the most significant number of respondents were female (62.5%), as was the case with other surveys [6,18,26,30,38,39,40,41,42,43,44]. Women are more likely to respond to online-type surveys than men, and women are known to have healthier eating habits than men [2,18,45,46].

For the 43 items (100%) that make up the UFG, the frequency of responses indicated as “True” varied between 3.8 and 99.2%. The frequency of responses indicated as “False” varied between 0.7 and 92.3%, while “I don’t know” varied between 0.1 and 36.9% (Appendix C). Foods of animal origin, such as dairy products and some types of game, as well as some items of vegetable origin (coconut, avocado, lettuce, arugula, and kale), oils, and fats (animal and vegetable origin) were the items indicated as “True” by respondents. Grouping foods according to their origin, as animal or vegetable, was one of the first strategies for classifying foods and, therefore, is a type of classification that is familiar to most consumers. Regarding origin, in some FBDGs, foods of animal origin are further separated into two groups: a group related to meat and eggs and a group related to milk and dairy products, most likely because they are, respectively, sources of iron and calcium [16].

To prevent respondents from having doubts about the meaning of the terms “food classification” and “food groups”, the instrument was constructed from items (I-1—Meat is any edible part of animals/Appendix A), and the responses were grouped by researchers into their respective groups. This is because foods can be classified according to different criteria (origin, nature, processing, nutrient sources, food groups, etc.) [6,34].

According to a study previously carried out by Monteiro et al. [6] and the present study, most respondents (48.9%) believe it is easier to classify foods according to food groups. However, we identified that among the FBDGs researched, those that use food classification based on food groups do not present a logical composition since items such as green banana, coconut, avocado, dairy products, and legumes were indicated in different food groups, which have different nutritional and botanical standards [17].

A critical divergence was observed in the grouping of species in the plant kingdom. From the point of view of edible parts, vegetables are classified as roots, tubers, rhizomes, leaves, fruits, tender stalks and shoots, inflorescences/flowers, bulbs, immature seeds, and cultivated mushrooms. Given this, lettuce, kale, chicory, cabbage, watercress, and spinach are leaves; yam, cara, and potato are tubers, while taro is a rhizome. Onions and garlic are bulbs; cauliflower, broccoli, and artichokes are inflorescences/flowers;, zucchini, okra, eggplant, green corn, tomatoes, pumpkins, melons, watermelons, and strawberries are fruits. Snap beans, green beans, peas, and snow peas are immature pods and seeds. Carrots, cassava, beets, radishes, turnips, sweet potatoes, yacon, and parsnips are roots, while celery/celery, asparagus, and celtuce are tender stalks. From a nutritional point of view, edible parts such as tubers, roots, and rhizomes contain a higher level of energy reserves (carbohydrates) [47]. On the other hand, snap and green beans can have up to 40% protein in their chemical composition, depending on the variety. Fruits, leaves, and bulbs are sources of vitamins, minerals, and bioactive compounds, such as flavonoids.

As for cereals and legumes, it is also essential to consider their chemical properties to estimate their nutritional contribution. These crops are grain/seed suppliers. Grains/seeds, such as rice, wheat, corn, and their derivatives, are sources of complex carbohydrates. Legumes, such as mature beans, lentils, broad beans, chickpeas, peanuts, and soybeans, are sources of proteins and carbohydrates [18,48]. Oilseeds are plants that contain a high oil content, both from their seeds (soybean, rapeseed/canola, sunflower, castor, jatropha, crambe, chia) and from their fruits (palm, babassu, coconut). Soybeans, considering protein content, can be grouped as legumes; however, if the parameter is industrial processing, soybeans are classified as oilseeds (sources of fats) [49]. The results suggest that Brazilian respondents indicated item 22 (nuts, chestnuts, and seeds are “legumes” or vegetables) as “False” (*n* = 825; 92.3%) since for more than 60% of them, beans, soy, lentils, chickpeas are understood as legumes or legume seeds. Beans and other legumes are foods that, according to the FBDGs, can be classified in the fruit and vegetable group, the cereals group, or even included in the meat group or with oilseeds [17,34]. As we already highlighted, these foods differ in chemical composition and nutritional properties.

A study by Reyneke et al. [18] evaluated 314 Australian respondents’ comprehension of the terms used for the items “pulses and whole grains” described in their country’s dietary guidelines. The results showed that the majority (*n* = 123; 45%) of respondents indicated that “legumes” should be in their group or as part of the protein source food group. According to the Codex Alimentarius [50], pulses, nuts, and seeds are included in a specific group.

According to the results, more than 90% of the Brazilian respondents understand item 24 (seafood, poultry, vegetables, eggs, and dairy products are meat) as “False” (*n* = 807; 90.3%), which suggests that, for this sample of Brazilians, legumes are not meat (I-24). More than 80% of the Brazilian respondents indicated item 14 (soy steak is a type of meat) as “False” (*n* = 751; 84%). Therefore, considering results and data from previous studies on understanding the group that legumes should be part of, as well as the nutritional and botanical characteristics of these food items, the FBDGs must create a specific group for items that belong to the legume group.

Understanding relates to the consensus that is acquired about an event or issue. Knowing does not mean understanding, despite being closely linked. Understanding refers to interpreting, evaluating, or perceiving what is being treated, discussed, and particularly exposed. These are essential to estimate the attribute’s value related to a cause [51,52,53,54]. The inability to understand something about what is in question can lead the consumer to make mistakes in their judgment. In this way, various proposals to classify foods have emerged, as well as food classification systems aiming to associate food groups or eating patterns with sociodemographic data and diseases. In the literature, few studies on knowledge/understanding about food groups and sociodemographic data have been published [6,18,26,27,28,55], although several studies evaluate the consumption of a certain food group with sociodemographic data and diseases [56,57,58,59,60,61,62,63,64,65,66].

Riedeiger et al. [59] reported that the female gender, family education, and high income positively impacted adolescents’ fruit and vegetable consumption in Canada. In this study, male respondents from the Central-west region indicated a higher percentage of responses as “True” (59.4%) for item I-10 (vegetables are leaves, flowers, fruits, stems, seeds, tubers, and roots). Costa et al. [56] evaluated the food consumption of Brazilian adults in urban and rural areas and concluded that in the Northeast macro-region, there was lower consumption of fruits and vegetables compared with the consumption in other Brazilian regions. Fruits are essential to a healthy diet because they contain vitamins and minerals, fiber, and beneficial non-nutrient substances such as bioactive compounds. The World Health Organization (WHO) recommends ingesting at least 400 g (about five portions) of fruits and vegetables daily. Low fruit consumption is one of the main risk factors for increased mortality and also increases the risk of chronic diseases and poor health quality [4,60,67].

Brazilian respondents from the Southeast region indicated a higher percentage of “True” responses for I-7 (tomato, melon, watermelon, pepper, and bell pepper are fruits) (87.6%). The Southeast region is responsible for 40.87% of Brazil’s fruit and vegetable production [68]. In botanical terms, a tomato is a fruit. Consumers generally recognize this food item as “verdura” [69,70].

Baek and Chitekwe [71] studied the food consumption of children and adolescents in Nepal and observed differences in the consumption of various foods, including dairy products, according to geographic location and income. In this study, for the geographic region criterion, we identified a significant difference for items I-8, I-13, and I-23. According to Zoccal [72], the North region is the one that consumes the least amount of milk, which could explain why respondents from the North region indicated a higher percentage of “True” for items I-13 (soy milk and rice milk are milk) and I-23 (tofu is a cheese).

Guine et al. [73] evaluated dairy product consumption habits in Brazilian and Portuguese adults. They found that semi-skimmed milk was never consumed by about half of the Brazilian respondents (46.7%), and this number increased for skimmed milk (50.9%), chocolate-flavored milk (65.6%), and enriched milk (85.3%). The number of participants consuming imported cheeses in both countries was particularly low (only 4.0% consumed these items more than once a week), suggesting that national products may be preferred. They also found that those who consume cheese do so rarely (once a week) or sometimes (two to three times a week). Yogurt consumption also follows the same trend toward low consumption. The most consumed types of yogurts in Brazil are creamy fruit pulp yogurts (14.4% consume regularly), liquid yogurts (13.7% consume regularly), and Greek-type yogurt (10.2% consume regularly). Guine et al. [73] concluded that, despite some slight differences in dairy product consumption patterns, in both countries (Brazil and Portugal), dairy product consumption levels were extremely low for all evaluated items (milk, cheese, yogurt, and butter).

Costa et al. [56] verified differences in fish consumption among Brazilian macro-regions and identified greater consumption in the Northern and Northeast regions. In this study, respondents from the Northeast region indicated a higher percentage of “True” responses (9.6%, 6.4% overall) for item I-9 (seaweed is a seafood). This means that more than 80% of respondents understand that algae are not seafood or do not understand that algae can be consumed as a food despite the maritime extension of the Northeast region being the largest in the country, which may represent greater availability of algae [74]. Around 13% of respondents indicated that they did not know how to answer item I-9, regardless of the region.

The results identified that respondents from the South region indicated I-36 (beans, soy, lentils, and chickpeas are legumes) (73%) and I-41 (beans of all colors, fava beans, lentils, and peanuts are legume seeds) (85%) as “True”. Costa et al. [56] also observed that adults in the Southeast and Central-west regions consume more beans, justifying the relationship between understanding and consumption.

Regarding the food items included in the “meat group” or “meat product group”, Brazilian respondents indicated a higher percentage of “True” responses for item I-5 (hot dogs, sausage, ham, and mortadella, among others, are meat products) than for item I-21 (hot dogs, sausage, ham, and mortadella, among others, are meats). Meat and meat products/derivatives have very different chemical and nutritional composition. While meat cuts from different species differ wildly in terms of lipid content, meat derivatives have different values of sodium and, possibly, saturated fat content, depending on the industrial formulation. Thus, the recommendation for meat products (mortadella, sausage, salami, ham, and bacon) must be evaluated in terms of quantity and frequency of consumption [75,76,77] in the FBDGs because their consumption is associated with NCD risk.

We also observed that foods such as insects, kefir, and green bananas are little-consumed and little-known by Brazilian respondents in this study [78,79,80,81,82]. The FBDGs of Vietnam, Korea, Cambodia, and Kenya included the consumption of insects [17,34]. For FAO, all terrestrial insects such as spiders, mites, ticks, beetles, flies, bugs, and ants, and grubs such as earthworms, including processed products such as dried insects and manufactured products such as powdered insects and grubs, are defined in the nutrition subgroup “Insects and grubs” [50]. More than 60% (*n* = 544) of Brazilian respondents indicated item I-17 (insects such as grasshoppers, ants, grubs, cicadas, and dragonflies are meat) as “False”, which suggests that insects are not considered to be meat by this sample of Brazilians. Furthermore, around 18% (*n* = 159) of respondents indicated “I do not know” for this same item.

Kefir is a fermented drink with low alcohol content and is acidic and bubbly from the fermentation carbonation of kefir grains with milk or water [83]. In the FBDGs of Colombia, Estonia, and Hungary, kefir was included in the dairy products group [17,34]. Around 38% (*n* = 338) of Brazilian respondents indicated item I-8 (kefir is a dairy product) as “True”, 25% (*n* = 226) indicated it as “False”, and 37% (*n* = 330) indicated it as “I do not know”. Women were those who indicated the highest percentage of “True” responses, as well as respondents from the South region. Female respondents are most concerned about health and aspects linked to the nutritional value of food [2,6,18,45,46].

The FBDGs of 17 Latin American countries added green bananas to the cereals and pulses group [17,34]. Around 87% (*n* = 774) of the respondents indicated I-2 (green banana is a type of cereal) as “False”. Respondents with an education level of up to high school and an income of up to four minimum wages indicated the highest percentage of “True” responses for I-2. These data may suggest that respondents with lower education and income in this study’s sample understand that green bananas are a type of cereal, while for respondents from other sociodemographic groups, green bananas are included in the fruit group. Green bananas seem to be a good source of fiber, vitamins, bioactive compounds such as phenolic compounds, and resistant starch (RS), potentially contributing to health benefits [67,84].

The results suggest that women indicated a higher percentage of items as “True” for items referring to seasonings (I-6: garlic and onions are bulbs, 81%), culinary preparations with plant extracts (I-13: soy milk and rice milk are milk, 38.6%; I-23: tofu is a cheese, 44.4%), culinary preparations with milk (I-8: kefir is a dairy product, 42.4%; I-19: curd is a dairy product, 98.9%), and meat (I-31: rabbit, wild boar, snake, and alligator meats are game meats, 93%; I-1: meat is any edible part of animals, 46%). Studies on food choices show that, in general, women are responsible for preparing meals at home or the family’s food choices, thus justifying the higher percentage of “True” responses for the items highlighted above [85,86,87,88].

Some of the limitations of this study are possibly due to the instrument’s application type. The online system via social media limits the sample to the group of consumers with access to the Internet (a global system of interconnected computer networks that use their own set of protocols—Internet Protocol Suite or TCP/IP). Thus, for the consumer to participate in this research, they must have access to the Internet, be literate, and be interested in issues relating to food versus nutrition. According to the data obtained, our sample contained a greater number of female respondents with a postgraduate education level and with income greater than 10 minimum wages.

## 5. Conclusions

The present work provided a valuable international overview of 89 FBDGs available on the .FAO website. The data indicate that consumers believe it is easier to classify foods according to food groups. However, although traditionally, consumers easily recognize foods according to their origin—animal or vegetable—we still identified asymmetries regarding the inclusion of food items in the group of species from the animal kingdom and in the group of species from the plant kingdom.

It is worth highlighting that the improper grouping of food items can lead to mistaken interpretations when studies involve the relationship between food consumption and health.

We conclude that this exploratory study highlights important information that can contribute to improving the dietary guidelines presented in the FBDGs. It is essential to consider consumers’ understanding and guide them regarding choices from a technical point of view. Why? Because food is an important source of nutrients for maintaining our vital activities, and a balance between the quantity and quality of nutrients accounts for the best quality of health. Therefore, it is essential to disseminate correct information, always considering cultural characteristics, socioeconomic characteristics, and educational levels. Therefore, the food items described in the FBDGs should be redistributed into their respective groups so that the differences already widely identified in studies on food consumption and their contribution to health are minimized. We identified the need for new studies in other countries that evaluate consumer understanding of food groups, not just a particular group or food consumption, with the aim of improving consumer adherence to dietary guidelines.

## Figures and Tables

**Table 1:** The numbers and frequency of responses indicated by respondents as “True” with a frequency greater than 90%.

Items	True		False		I Do not Know	
	*n*	%	*n*	%	*n*	%
I-15. Yogurt, cheeses, and cottage cheese are dairy products (“produtos lácteos”).	882	98.7	10	1.1	2	0.2
I-18. Sweet potatoes, carrots, cassava, and turnips are roots.	832	93.1	48	5.4	14	1.6
I-19. Curd is a dairy product.	879	98.3	6	0.7	9	1
I-20. Coconut and avocado are fruits.	823	92.1	45	5	26	2.9
I-31. Rabbit, wild boar, snake, and alligator meat are game meats.	808	90.4	59	6.6	27	3
I-32. Yogurt, cheese, and cottage cheese are derived from milk.	887	99.2	6	0.7	1	0.1
I-37. Lettuce, arugula, and kale are leafy foods.	865	96.8	12	1.3	17	1.9
I-40. Yogurt, cheeses, and cottage cheese are dairy products (“laticínios”)	843	94.3	37	4.1	14	1.6
I-42. Oils, olive oils, butter, and margarine are fats and oils.	842	94.2	32	3.6	20	2.2

**Table 2:** Consumer understanding of UFG items included in the animal groups and sociodemographic data.

Items Included in the Animal Groups	Characteristics	Category	% True	*p*-Value
Meat is any edible part of animals (I-1)	Gender	Male	36.4	*p* = 0.00 *p* < 0.05
		Female	46	
Rabbit, wild boar, snake, and alligator meat are game meats (I-31)		Male	86	*p* = 0.02 *p* < 0.05
		Female	93	
Meat is beef (I-11)	Educational level	Up to high school	14	*p* = 0.00 *p* < 0.05
		College degree	19	
		Postgraduation	11.1	
Seafood, poultry, legumes, eggs, and dairy products are meat (I-24)		Up to high school	11.6	
		College degree	10.7	
		Postgraduation	5.4	
Meat is beef (I-11)	Income—minimum wage (BRL 1100) USD 1 = BRL 5.16	Up to four	18.5	*p* = 0.023 *p* < 0.05
		From five to nine	12.3	
		Above 10	9.6	
		Not informed	11.6	
Seafood, poultry, legumes, eggs, and dairy products are meat (I-24)		Up to four	9	*p* = 0.007 *p* < 0.05
		From five to nine	6.9	
		Above 10	4.8	
		Not informed	9.3	
Meats are classified into beef, pork, sheep, goat, buffalo, poultry, fish, amphibians, mammals, and reptiles (I-43)	Age	Up to 39	62.8	*p* = 0.018 *p* < 0.05
		Over 40	70.6	
Seaweed is a seafood (I-9)	Region	Center-west	8.5	*p* = 0.05 *p* ≤ 0.05
		Northeast	9.6	
		North	6.7	
		Southeast	4.3	
		South	4	
	Educational level	Up to high school College degree	4.7 12.5	*p* = 0.00 *p* < 0.05
		Postgraduation	5	
	Income—minimum wage (BRL 1100) USD 1 = BRL 5.16	Up to four	10.8	*p* = 0.00 *p* ≤ 0.05
		From five to nine	5.1	
		Above 10	5.4	
		Not informed	0	
Fish are saltwater and freshwater fish (I-34)	Age	Up to 39	81.4	*p* = 0.004 *p* < 0.05
		Over 40	89	
	Income—minimum wage (BRL 1100) USD 1 = BRL 5.16	Up to four	83.3	*p* = 0.05 *p* < 0.05
		From five to nine	85.5	
		Above 10	87.3	
		Not informed	90.7	
Hot dogs, sausage, ham, and mortadella, among others, are meat derivatives (I-5)	Gender	Male	91.3	*p* = 0.025 *p* < 0.05
		Female	85.9	
	Age	Up to 39	84.2	*p* = 0.008 *p* < 0.05
		Over 40	90.5	
	Region	Center-west	80.5	*p* = 0.00 *p* < 0.05
		Northeast	81.7	
		North	89.2	
		Southeast	92.2	
		South	93	
Hot dogs, sausage, ham, and mortadella, among others, are meats (I-21)	Gender	Male	50.7	*p* = 0.021 *p* < 0.05
		Female	41.3	
	Region	Center-west	40.7	*p* = 0.005 *p* < 0.05
		Northeast	34.1	
		North	45	
		Southeast	50.9	
		South	51	
Kefir is milk-derived (I-8)	Gender	Male	30.1	*p* = 0.00 *p* < 0.05
		Female	42.4	
	Age	Up to 39	37.4	*p* = 0.05 *p* ≤ 0.05
		Over 40	38.1	
	Region	Center-west	39	*p* = 0.00 *p* < 0.05
		Northeast	28.4	
		North	40	
		Southeast	39.7	
		South	47	
Soy milk and rice milk are milk (I-13)	Gender	Male	26.9	*p* = 0.002 *p* < 0.05
		Female	38.6	
	Age	Up to 39	39.3	*p* = 0.008 *p* < 0.05
		Over 40	30.7	
	Region	Center-west	39	*p* = 0.015 *p* < 0.05
		Northeast	38.5	
		North	45	
		Southeast	27	
		South	32	
	Educational level	Up to high school	37.2	*p* = 0.024 *p* < 0.05
		College degree	43.5	
		Postgraduation	31.8	
	Income—minimum wage (BRL 1100) USD 1 = BRL 5.16	Up to four	44.1	*p* = 0.00 *p* < 0.05
		From five to nine	35.1	
		Above 10	27.8	
		Not informed	34.2	
Curd is a dairy product (I-19)	Gender	Male	97.3	*p* = 0.042 *p* < 0.05
		Female	98.9	
	Age	Up to 39	96.7	*p* = 0.006 *p* < 0.05
		Over 40	99.4	
	Educational level	Up to high school	100	*p* = 0.00 *p* < 0.05
		College degree	94.6	
		Postgraduation	99.1	
	Income—minimum wage (BRL 1100) USD 1 = BRL 5.16	Up to four	96.4	*p* = 0.026 *p* < 0.05
		From five to nine	98.9	
		Above 10	98.9	
		Not informed	100	
Tofu is a cheese (I-23)	Gender	Male	37.6	*p* = 0.00 *p* < 0.05
		Female	44.4	
	Age	Up to 39	36.9	*p* = 0.041 *p* < 0.05
		Over 40	45.3	
	Region	Center-west	49.2	*p* = 0.00 *p* < 0.05
		Northeast	48.1	
		North	50	
		Southeast	32.8	
		South	42	
Yogurt, cheeses, and cottage cheese are dairy products (I-40)	Age	Up to 39	91.8	*p* = 0.028 *p* < 0.05
		Over 40	96	
	Educational level	Up to high school	83.7	*p* = 0.006 *p* < 0.05
		College degree	92.3	
		Postgraduation	95.5	

**Table 3:** Consumer understanding of UFG items included in the vegetal groups and sociodemographic data.

Items Included in the Vegetal Groups	Characteristics	Category	% True	*p*-Value
Green banana is a type of cereal (I-2)	Educational level	Up to high school	9.3	*p* = 0.001 *p* < 0.05
		College degree	6	
		Postgraduation	3.5	
	Income—minimum wage (BRL 1100) USD 1 = BRL 5.16	Up to four	6.8	*p* = 0.006 *p* < 0.05
		From five to nine	4	
		Above 10	2.8	
		Not informed	4.7	
Oats, rice, wheat, and corn are cereal seeds (I-12)	Gender	Male	90.4	*p* = 0.009 *p* < 0.05
		Female	83	
Gnocchi, pasta, lasagna, cannelloni are derived from cereals (I-35)		Male	62.4	*p* = 0.011 *p* < 0.05
		Female	52.2	
Corn, as grain or on the cob, wheat grains, and other cereals are part of the group of cereals and legumes (I-39)		Male	51.6	*p* = 0.011 *p* < 0.05
		Female	58.9	
Soy and chickpeas are vegetables (“legumes”) (I-33)	Gender	Male	31	*p* = 0.035 *p* < 0.05
		Female	24.5	
	Age	Up to 39	21.6	*p* = 0.007 *p* < 0.05
		Over 40	30.7	
Soy steak is a type of meat (I-14)	Region	Center-west	25.4	*p* = 0.000 *p* < 0.05
		Northeast	16.8	
		North	18.3	
		Southeast	8	
		South	3	
	Educational level	Up to high school	14	*p* = 0.000 *p* < 0.05
		College degree	24.4	
		Postgraduation	10.4	
	Income—minimum wage (BRL 1100) USD 1 = BRL 5.16	Up to four	21.2	*p* = 0.000 *p* < 0.05
		From five to nine	13.4	
		Above 10	8.8	
		Not informed	7	
Beans, soy, lentils, and chickpeas are cereals (I-16)	Gender	Male	52.2	*p* = 0.013 *p* < 0.05
		Female	48.1	
	Age	Up to 39	42.3	*p* = 0.001 *p* < 0.05
		Over 40	54.7	
Beans, soybeans, lentils, and chickpeas are vegetables (I-27)	Gender	Male	66.3	*p* = 0.000 *p* < 0.05
		Female	53.1	
	Age	Up to 39	48.9	*p* = 0.000 *p* < 0.05
		Over 40	64.4	
	Educational level	Up to high school	44.2	*p* = 0.002 *p* < 0.05
		College degree	47	
		Postgraduation	61.6	
	Income—minimum wage (BRL 1100) USD 1 = BRL 5.16	Up to four	47.7	*p* = 0.01 *p* < 0.05
		From five to nine	60.5	
		Above 10	61.8	
		Not informed	65.1	
Beans, soy, lentils, chickpeas are legumes (I-36)	Region	Center-west	71.2	*p* = 0.016 *p* < 0.05
		Northeast	62.5	
		North	60.8	
		Southeast	71.6	
		South	73	
	Educational level	Up to high school	51.2	*p* = 0.013 *p* < 0.05
		College degree	60.7	
		Postgraduation	71	
	Income—minimum wage (BRL 1100) USD 1 = BRL 5.16	Up to four	62.2	*p* = 0.025 *p* < 0.05
		From five to nine	66.3	
		Above 10	73.9	
		Not informed	62.8	
Beans of all colors, fava beans, lentils, and peanuts are legume seeds (I-41)	Age	Up to 39	69.9	*p* = 0.025 *p* < 0.05
		Over 40	77.7	
	Region	Center-west	72	*p* = 0.003 *p* < 0.05
		Northeast	73.6	
		North	70	
		Southeast	74.4	
		South	85	
Cauliflower, broccoli, and artichoke are flowers (I-4)	Age	Up to 39	56	*p* = 0.003 *p* < 0.05
		Over 40	67	
	Educational level	Up to high school	79.1	*p* = 0.004 *p* < 0.05
		College degree	51.2	
		Postgraduation	64.3	
Garlic and onions are bulbs (I-6)	Gender	Male	74.6	*p* = 0.007 *p* < 0.05
		Female	81	
	Age	Up to 39	69.1	*p* = 0.00 *p* < 0.05
		Over 40	85.2	
	Region	Center-west	83.1	*p* = 0.019 *p* < 0.05
		Northeast	76.9	
		North	72.5	
		Southeast	78.7	
		South	84	
	Income—minimum wage (BRL 1100) USD 1 = BRL 5.16	Up to four	70.3	*p* = 0.014 *p* < 0.05
		From five to nine	78.6	
		Above 10	83	
		Not informed	86	
Tomato, melon, watermelon, pepper, bell peppers are fruits (I-7)	Region	Center-west	78	*p* = 0.021 *p* < 0.05
		Northeast	84.1	
		North	81.7	
		Southeast	87.6	
		South	84	
Vegetables are leaves, flowers, fruits, stems, seeds, tubers, and roots (I-10)	Gender	Male	59.4	*p* = 0.024 *p* < 0.05
		Female	54.2	
	Region	Center-west	69.5	*p* = 0.035 *p* < 0.05
		Northeast	54.3	
		North	53.3	
		Southeast	51.7	
		South	63	
Sweet potatoes, carrots, cassava, and turnips are roots (I-18)	Educational level	Up to high school	88.4	*p* = 0.003 *p* < 0.05
		College degree	91.1	
		Postgraduation	93.9	
Nuts, chestnuts, and seeds are vegetables (I-22)	Age	Up to 39	1.1	*p* = 0.001 *p* < 0.05
		Over 40	5.7	
	Educational level	Up to high school	2.3	*p* = 0.001 *p* < 0.05
		College degree	1.2	
		Postgraduation	4.5	
“*Verduras*” are the edible green (leafy) parts of plants (I-25)	Age	Up to 39	47	*p* = 0.00 *p* < 0.05
		Over 40	66.7	
	Region	Center-west	50.8	*p* = 0.00 *p* < 0.05
		Northeast	44.7	
		North	49.2	
		Southeast	71.3	
		South	64	
	Educational level	Up to high school	53.5	*p* = 0.016 *p* < 0.05
		College degree	47.6	
		Postgraduation	61.6	
	Income—minimum wage (BRL 1.100) USD 1 = BRL 5.16	Up to four	51.4	*p* = 0.025 *p* < 0.05
		From five to nine	56.9	
		Above 10	64	
		Not informed	62.8	
Lettuce, arugula, and kale are leafy foods (I-37)	Age	Up to 39	96.2	*p* = 0.035 *p* < 0.05
		Over 40	97.2	

## Data Availability

The data are contained within this article and the Appendix A, Appendix B and Appendix C, further inquiries can be directed to the corresponding author.

## Appendix A. Items from the Understanding of Food Groups (UFG) Instrument

**Table A1:** For each item below, mark True, for true alternatives, False, for false alternatives, or I don’t know, for alternatives that you cannot judge.

Meat is any edible part of animals. (Em português brasileiro)—I-1. Carne é qualquer parte comestível dos animais)		
True ( )	False ( )	I don’t know ( )
Green banana is a type of cereal. (Em português brasileiro)—I-2. Banana verde é um tipo de cereal)		
True ( )	False ( )	I don’t know ( )
Dry curd is milk derived. (Em português brasileiro)—I-3. A coalhada seca é derivada do leite).		
True ( )	False ( )	I don’t know ( )
Cauliflower, broccoli, artichoke are flowers. (Em português brasileiro)—I-4. Couve-flor, brócolis, alcachofra são flores).		
True ( )	False ( )	I don’t know ( )
Hot dogs, sausage, ham, mortadella, among others, are meat derivatives. (Em português brasileiro)—I-5. Salsicha, linguiça, presunto, mortadela, entre outros, são derivados cárneos)		
True ( )	False ( )	I don’t know ( )
Garlic and onion are bulbs. (Em português brasileiro)—I-6. Alho e cebola são bulbos)		
True ( )	False ( )	I don’t know ( )
Tomato, melon, watermelon, pepper, bell pepper are fruits. (Em português brasileiro)—I-7. Tomate, melão, melancia, pimenta, pimentão são frutos)		
True ( )	False ( )	I don’t know ( )
Kefir is milk derived. (Em português brasileiro)—I-8. Kefir é derivado do leite).		
True ( )	False ( )	I don’t know ( )
Seaweed is a seafood. (Em português brasileiro)—I-9. Algas são carnes de pescados).		
True ( )	False ( )	I don’t know ( )
Vegetables are leaves, flowers, fruits, stems, seeds, tubers and roots. (Em português brasileiro)—I-10. Hortaliças são folhas, flores, frutos, caules, sementes, tubérculos e raízes).		
True ( )	False ( )	I don’t know ( )
Meat is beef. (Em português brasileiro)—I-11. Carne é carne de boi).		
True ( )	False ( )	I don’t know ( )
Oats, rice, wheat, corn are cereal seeds. (Em português brasileiro)—I-12. Aveia, arroz, trigo, milho são sementes de cereais).		
True ( )	False ( )	I don’t know ( )
Soy milk and rice milk are milks. (Em português brasileiro)—I-13. Leites de soja e de arroz são leites).		
True ( )	False ( )	I don’t know ( )
Soy steak is a type of meat. (Em português brasileiro)—I-14. O bife de soja é um tipo de carne)		
True ( )	False ( )	I don’t know ( )
Yogurt, cheeses, cottage cheese are dairy products (“*produtos lácteos*”). (Em português brasileiro)—I-15. Iogurte, queijos, requeijão são produtos lácteos).		
True ( )	False ( )	I don’t know ( )
Beans, soy, lentils, chickpeas are cereals. (Em português brasileiro)—I-16. Feijão, soja, lentilha, grão-de-bico são cereais).		
True ( )	False ( )	I don’t know ( )
Insects such as grasshoppers, ants, grubs, cicadas, dragonflies are meat. (Em português brasileiro)—I-17. Insetos como gafanhotos, formigas, larvas, cigarras, libélulas são carnes).		
True ( )	False ( )	I don’t know ( )
Sweet potatoes, carrots, cassava, turnips are roots. (Em português brasileiro)—I-18. Batata-doce, cenoura, mandioca, nabo são raízes).		
True ( )	False ( )	I don’t know ( )
Curd is a derivative of milk. (Em português brasileiro)—I-19. Coalhada é um derivado do leite).		
True ( )	False ( )	I don’t know ( )
Coconut and avocado are fruits. (Em português brasileiro)—I-20. Coco e abacate são frutas).		
True ( )	False ( )	I don’t know ( )
Hot dogs, sausage, ham, mortadella, among others, are meats. (Em português brasileiro)—I-21. Salsicha, linguiça, presunto, mortadela, entre outros, são carnes).		
True ( )	False ( )	I don’t know ( )
Nuts, chestnuts, seeds are *“legumes”*. (Em português brasileiro)—I-22. Nozes, castanhas, sementes são legumes).		
True ( )	False ( )	I don’t know ( )
Tofu is a cheese. (Em português brasileiro)—I-23. Tofu é um queijo).		
True ( )	False ( )	I don’t know ( )
Seafood, poultry, legumes, eggs, dairy products are meat. (Em português brasileiro)—I-24. Pescados, aves, leguminosas, ovos, lácteos são carnes).		
True ( )	False ( )	I don’t know ( )
*“Verduras”* are the edible green (leafy) parts of plants. (Em português brasileiro)—I-25. Verduras são as partes verdes (folhosos) comestíveis das plantas).		
True ( )	False ( )	I don’t know ( )
Flour, tapioca, starch (*maisena*®), tapioca are cereals. (Em português brasileiro)—I-26. Farinhas, tapioca, amido (maisena), polvilho são cereais).		
True ( )	False ( )	I don’t know ( )
Beans, soybeans, lentils, chickpeas are vegetables. (Em português brasileiro)—I-27. Feijão, soja, lentilha, grão-de-bico são vegetais).		
True ( )	False ( )	I don’t know ( )
Viscera (offals), blood, fat, cartilage, bones are meat. (Em português brasileiro)—I-28. Vísceras (miúdos), sangue, gordura, cartilagens, ossos são carnes).		
True ( )	False ( )	I don’t know ( )
Starchy products, such as flour, tapioca, starch, tapioca, are natural. (Em português brasileiro)—I-29. Vísceras (miúdos), sangue, gordura, cartilagens, ossos são carnes).		
True ( )	False ( )	I don’t know ( )
Potato, beet, chayote are vegetables. (Em português brasileiro)—I-30. Batata, beterraba, chuchu são legumes).		
True ( )	False ( )	I don’t know ( )
Rabbit, wild boar, snake, alligator meat are game meats. (Em português brasileiro)—I-31. Batata, beterraba, chuchu são legumes).		
True ( )	False ( )	I don’t know ( )
Yogurt, cheese, cottage cheese are derived from milk. (Em português brasileiro)—I-32. Iogurte, queijos, requeijão são derivados do leite).		
True ( )	False ( )	I don’t know ( )
Soy and chickpeas are *“legumes”*. (Em português brasileiro)—I-33. Soja e grão-de-bico são legumes).		
True ( )	False ( )	I don’t know ( )
Fished are saltwater and freshwater fish. (Em português brasileiro)—I-34. Pescados são peixes de água salgada e de água doce).		
True ( )	False ( )	I don’t know ( )
Gnocchi, pasta, lasagna, cannelloni are derived from cereals. (Em português brasileiro)—I-35. Inhoque, macarrão, lasanha, canelone são derivados de cereais).		
True ( )	False ( )	I don’t know ( )
Beans, soy, lentils, chickpeas are legumes. (Em português brasileiro)—I-36. Feijão, soja, lentilha, grão-de-bico são leguminosas).		
True ( )	False ( )	I don’t know ( )
Lettuce, arugula and kale are leafy foods. (Em português brasileiro)—I-37. Alface, rúcula e couve são alimentos folhosos.		
True ( )	False ( )	I don’t know ( )
White meats are poultry and fish. (Em português brasileiro)—I-38. Carnes brancas são as de aves e de pescados).		
True ( )	False ( )	I don’t know ( )
Corn, in grain or on the cob, wheat grains and other cereals are part of the group of cereals and legumes. (Em português brasileiro)—I-39. Milho, em grão ou na espiga, grãos de trigo e de outros cereais fazem parte do grupo de cereais e legumes.		
True ( )	False ( )	I don’t know ( )
Yogurt, cheeses, cottage cheese are dairy products (“*laticínios*”). (Em português brasileiro)—I-40. Iogurte, queijos, requeijão são laticínios).		
True ( )	False ( )	I don’t know ( )
Beans of all colors, fava beans, lentils and peanuts are legume seeds. (Em português brasileiro)—I-41. Feijão de todas as cores, fava, lentilha e amendoim são sementes de leguminosas).		
True ( )	False ( )	I don’t know ( )
Oils, olive oils, butters, margarines are fats and oils. (Em português brasileiro)—I-42. Óleos, azeites, manteigas, margarinas são gorduras e óleos.		
True ( )	False ( )	I don’t know ( )
Meats are classified into beef, pork, sheep, goat, buffalo, poultry, fish, amphibians, mammals, reptiles. (Em português brasileiro)—I-43. Carnes são classificadas em bovina, suína, ovina, caprina, bubalina, aves, pescados, anfíbios, mamíferos, répteis).		
True ( )	False ( )	I don’t know ( )

**Table A2:** Indicate how you find it easier to choose foods.

**How easy is it for you to choose foods:**
(a) Processing level”. Examples: *in natura*, minimally processed, processed, and ultra-processed. (b) Food groups. Examples: grains, meat, fruits and vegetables, and milk and eggs. (c) Nutrient sources. Examples: carbohydrate source, protein source, fat source, vitamin source, mineral source. (d) Ingredients list—the main ingredient, followed by the others (descending order).

## Appendix B. General Sociodemographic Data from the UFG Application

**Table A3:** National distribution of participants.

Region	Brazilian Population		Participants		
	(*n*)	(%)	(*n*)	(%)	Adequacy ≥ 70%
Center-west	16,287,809	8.02	118	13.2	164.58
Northeast	54,644,582	26.91	208	23.26	86.43
North	17,349,619	8.54	120	13.42	157.14
Southeast	84,847,187	41.78	348	38.92	93.15
South	29,933,315	14.74	100	11.18	75.84
OVERALL	203,062,512	100%	894	100%	All in accordance

**Table A4:** Sociodemographic data of Brazilian respondents in the UFG research, 2023.

Characteristics	Category	Respondents	
		N	(%)
Gender	Male	335	37.5
	Female	559	62.5
Age (years)	Up to 39	366	41
	Over 40	528	59
Educational level	Up to high school	43	4.8
	College degree	168	18.8
	Post-graduation	683	76.4
Income—minimum wage (BRL 1100) USD 1 = BRL 5.16	Up to four	222	24.8
	From five to nine	276	30.9
	Above 10	353	39.5
	Not informed	43	4.8

## Appendix C. Number and Frequency of Responses Indicated as “True”, “False”, and “I Do Not Know” for the Items Described in Block 1 of the Understanding Food Group

**Table:**

Number	Item	True		False		I Do Not Know	
		(*n*)	(%)	(*n*)	(%)	(*n*)	(%)
1.	Meat is any edible part of animals.	379	42.4	475	53.1	40	4.5
2.	Green banana is a type of cereal.	38	4.3	774	86.6	82	9.2
3.	Dry curd is milk-derived.	757	84.7	19	2.1	118	13.2
4.	Cauliflower, broccoli, and artichoke are flowers.	559	62.5	225	25.2	110	12.3
5.	Hot dogs, sausage, ham, and mortadella, among others, are meat products.	786	87.9	67	7.5	41	4.6
6.	Garlic and onions are bulbs.	703	78.6	30	3.4	161	18
7.	Tomato, melon, watermelon, pepper, and bell peppers are fruits.	754	84.3	94	10.5	46	5.1
8.	Kefir is milk-derived or Kefir is derived from milk.	338	37.8	226	25.3	330	36.9
9.	Seaweed is a seafood.	57	6.4	720	80.5	117	13.1
10.	Vegetables are leaves, flowers, fruits, stems, seeds, tubers, and roots.	502	56.2	303	33.9	89	10
11.	Meat is beef.	114	12.8	770	86.1	10	1.1
12.	Oats, rice, wheat, and corn are cereal seeds	767	85.8	84	9.4	43	4.8
13.	Soy milk and rice milk are milks.	306	34.2	514	57.5	74	8.3
14.	Soy steak is a type of meat.	118	13.2	751	84	25	2.8
15.	Yogurt, cheeses, and cottage cheese are dairy products (“produtos lácteos”)	882	98.7	10	1.1	2	0.2
16.	Beans, soy, lentils, and chickpeas are cereals.	444	49.7	419	46.9	31	3.5
17.	Insects such as grasshoppers, ants, grubs, cicadas, and dragonflies are meat.	191	21.4	544	60.9	159	17.8
18.	Sweet potatoes, carrots, cassava, and turnips are roots	832	93.1	48	5.4	14	1.6
19.	Curd is a derivative of milk	879	98.3	6	0.7	9	1
20.	Coconut and avocado are fruits.	823	92.1	45	5	26	2.9
21.	Hot dogs, sausage, ham, and mortadella, among others, are meats	401	44.9	456	51	37	4.1
22.	Nuts, chestnuts,and seeds are “legumes”.	34	3.8	825	92.3	35	3.9
23.	Tofu is a cheese.	374	41.8	380	42.5	140	15.7
24.	Seafood, poultry, legumes, eggs, and dairy products are meat	60	6.7	807	90.3	27	3
25.	“Verduras” are the edible green (leafy) parts of plants.	524	58.6	297	33.2	73	8.2
26.	Flour, tapioca, starch (maisena^®^), and tapioca are cereals.	259	29	528	59.1	107	12
27.	Beans, soy, lentils, and chickpeas are vegetables.	519	58.1	335	37.5	40	4.5
28.	Viscera (offals), blood, fat, cartilage, and bones are meat.	212	23.7	609	68.1	73	8.2
29.	Starchy products, such as flour, tapioca, starch, and tapioca, are natural.	537	60.1	251	28.1	106	11.9
30.	Potato, beet, and chayote are vegetables.	661	73.9	210	23.5	23	2.6
31.	Rabbit, wild boar, snake, and alligator meats are game meats.	808	90.4	59	6.6	27	3
32.	Yogurt, cheese, abd cottage cheese are derived from milk.	887	99.2	6	0.7	1	0.1
33.	Soy and chickpeas are “legumes” or soy and chickpeas are vegetables.	241	27	584	65.3	69	7.7
34.	Fish are saltwater and freshwater fish.	768	85.9	75	8.4	51	5.7
35.	Gnocchi, pasta, lasagna, and cannelloni are derived from cereals.	501	56	311	34.8	82	9.2
36.	Beans, soy, lentils, and chickpeas are legumes.	609	68.1	204	22.8	81	9.1
37.	Lettuce, arugula, and kale are leafy foods.	865	96.8	12	1.3	17	1.9
38.	White meats are poultry and fish.	787	88	84	9.4	23	2.6
39.	Corn, as grain or on the cob, wheat grains, and other cereals are part of the cereals and legumes group.	502	56.2	279	31.2	113	12.6
40.	Yogurt, cheeses, and cottage cheese are dairy products (“laticínios”)	843	94.3	37	4.1	14	1.6
41.	Beans of all colors, fava beans, lentils, and peanuts are legume seeds.	666	74.5	110	12.3	118	13.2
42.	Oils, olive oils, butters, and margarines are fats and oils.	842	94.2	32	3.6	20	2.2
43.	Meats are classified into beef, pork, sheep, goat, buffalo, poultry, fish, amphibians, mammals, and reptiles.	603	67.4	137	15.3	154	17.2
